# Optical and electrical properties of undoped and doped Ge nanocrystals

**DOI:** 10.1186/1556-276X-7-143

**Published:** 2012-02-20

**Authors:** Samaresh Das, Rakesh Aluguri, Santanu Manna, Rajkumar Singha, Achintya Dhar, Lorenzo Pavesi, Samit Kumar Ray

**Affiliations:** 1 Department of Physics and Meteorology, Indian Institute of Technology Kharagpur, Kharagpur 721302, India; 2 Dipartimento di Fisica, Laboratorio di Nanoscienze, Università di Trento, Via Sommarive 14, 38100 Povo (Trento), Italy

## Abstract

Size-dependent photoluminescence characteristics from Ge nanocrystals embedded in different oxide matrices have been studied to demonstrate the light emission in the visible wavelength from quantum-confined charge carriers. On the other hand, the energy transfer mechanism between Er ions and Ge nanocrystals has been exploited to exhibit the emission in the optical fiber communication wavelength range. A broad visible electroluminescence, attributed to electron hole recombination of injected carriers in Ge nanocrystals, has been achieved. Nonvolatile flash-memory devices using Ge nanocrystal floating gates with different tunneling oxides including SiO_2_, Al_2_O_3_, HfO_2_, and variable oxide thickness [VARIOT] tunnel barrier have been fabricated. An improved charge storage characteristic with enhanced retention time has been achieved for the devices using VARIOT oxide floating gate.

## Introduction

The development of silicon-based optoelectronics has attracted a lot of attention over the past decade [1,2]. The concept is based on integration of Si-based photonic components, in which light can be generated, waveguided, modulated, amplified, and detected with the advanced electronic components to realize monolithically integrated Si-based optoelectronic circuits. The study of Ge [3-5] and Si [6,7] nanostructures is motivated by the prediction that quantum confinement of carriers leads to efficient luminescence despite the indirect nature of the energy gaps. Germanium nanocrystals [NCs] have been found to exhibit visible luminescence at room temperature [3-5,8,9]. However, the mechanism of visible luminescence from Si and Ge nanocrystals is still disputed. Rare earth-doped semiconductors also have been shown to be of remarkably important for combining electronic devices with optical elements [10]. During the last several decades, the optical properties of erbium-doped semiconductor materials have been extensively studied due to the intra-4f ^4^*I*_13/2 _→ ^4^*I*_15/2 _transition (first excited state to the ground state of Er^3+ ^ion), which overlaps with the 1.54 μm wavelength of maximum transmission of silica-based optical fibers. Since Ge has higher electron and hole mobility, larger excitonic Bohr radius than Si [5] and is compatible with planar Si technology, efforts are being made to study the optical properties of Er-doped Ge nanostructures.

On the other hand, flash memory with nanocrystals floating gate has received much attention because of the high-speed write/erase operation, long retention time, and small device size [11]. Ge with a smaller band gap compared to Si is expected to improve the memory characteristics by inducing a higher valence band offset between the Si substrate and nanocrystals [12,13]. A thick tunnel barrier can guarantee a long retention time of the flash-memory device, but unfortunately, it slows down the programming speed. A thinner tunnel barrier will result faster programming speed but shorten the retention time. The use of a physically thicker high-permittivity oxide ensures good retention characteristics. On the other hand, thin-tunneling barriers due to the low equivalent oxide thickness allow high currents across the tunneling oxide at low control gate voltages during programming and erasing cycles [9,14-16]. For Ge nanocrystals embedded in a high dielectric constant [high-k] material, the electrostatic energy is much higher due to the difference in the static dielectric constant of SiO_2 _and high-k oxides [17]. In 2003, VARIOT structured tunnel oxide was reported by Govoreanu et al. [18] for the first time. Simulations and experimental results showed that a larger injected gate current density is possible for the memory devices with VARIOT structure tunnel barrier compared to memories with only a single-layered tunnel oxide [18,19].

In this paper, we report the size- and host matrix-dependent photoluminescence [PL] and electroluminescence [EL] characteristics of Ge nanocrystals. The systematic study demonstrated the origin of visible luminescence due to the quantum confinement of carriers. The temperature-dependent characteristics of 1.54 μm emission from Er-doped Ge nanocrystals are also presented. An improved charge storage characteristic for the nanocrystal in trilayer structure is reported using high-k Al_2_O_3 _and HfO_2_, as compared to conventional SiO_2_. The experimental results showed that a VARIOT tunnel stack is attractive as a replacement for the traditional single-layer tunnel barrier for improving the data retention and programming speed simultaneously.

## Experimental details

Ge nanocrystals embedded in different dielectric matrix were prepared by radio frequency magnetron sputtering (EDWARDS ESM 100 system, Sussex, UK). The structures used in this study were metal-insulator-semiconductor [MIS] capacitors with a dielectric stack consisting of Ge NCs sandwiched between tunneling and capping oxides. P-type (100) Si substrates with resistivity 7-14 Ω cm were initially cleaned by Piranha process followed by dipping in dilute HF for 1 min to remove the native oxide from the surface. The details of sample preparation can be found elsewhere [9,15]. The sample details studied here are given in Table 1. Er-doped Ge nanocrystals in Al_2_O_3 _matrix were grown on p-Si (100) substrates at 600°C under vacuum using pulsed-laser deposition (KrF excimer laser with wavelength 248 nm, energy 300 mJ, and pulse duration 20 ns). The sample was then annealed at 900°C in N_2 _atmosphere for 1 h to form the Ge nanocrystal and to disperse Er throughout the sample. High-resolution transmission electron microscopy [HRTEM] was carried out using a JEM 2100F (JEOL, Tokyo, Japan) field emission system with an operating voltage of 200 kV to probe the formation of Ge nanocrystals. Photoluminescence spectra of samples were recorded using a He-Cd laser as an excitation source operating at 325 nm with an output power density of 1.3 W/cm^2 ^and a TRIAX 320 monochromator (Wotol, 60 rue Waldeck Rousseau, Lyon, France)fitted with a Hamamatsu PMT (R-928, Hamamatsu Photonics, Hamamatsu City, Japan) tube and InGaAs detector (Hamamatsu Photonics). The electroluminescence signals were collected with a Spectra-Pro 2300i monochromator (Roper Scientific GmbH, Ottobrunn, Germany) coupled with nitrogen-cooled charge coupled device camera. The electrical properties of the samples were measured by a Keithley semiconductor parameter analyzer (4200-SCS, Keithley instruments, Cleveland, OH).

**Table 1:** Details of various samples deposited by radio frequency magnetron sputtering system

Sample name	Tunnel oxide (nm)		Middle layer (nm)	Cap oxide (nm)	Post-deposition treatment (°C for 30 min in N_2_)
'RS-1'	SiO_2_: ~5		SiGe: 15	SiO_2_: 25	Annealed at 800
'RS-2'	SiO_2_: ~5		SiGe: 15	SiO_2_: 25	Annealed at 900
'RS-3'	SiO_2_: ~5		SiGe: 15	SiO_2_: 25	Annealed at 1,000
'RA'	SiO_2_: ~5		Ge + Al_2_O_3_: 15	Al_2_O_3_: 25	Annealed at 900
'RF'	HfO_2_: ~5		Ge + HfO_2_: 15	HfO_2_: 25	Annealed at 900

'RFS'	SiO_2_: ~2.5	HfO_2_: ~ 5	Ge+HfO_2_: 15	HfO_2_: 25	Annealed at 900

'RA', 'RFS', and 'RS' are sample structures.

## Results and discussions

### TEM studies of Ge nanocrystals

**Figure 1:**
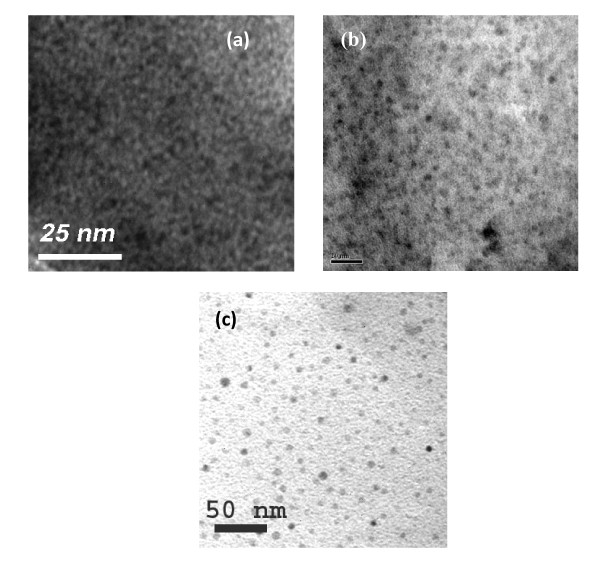
**Plane-view TEM micrograph of Ge nanocrystals embedded in SiO_2 _matrix for the sample**. (**a**) 'RS-1', (**b**) 'RS-2', and (**c**) 'RS-3'.

Figure 1 shows the TEM image of Ge nanocrystals embedded in SiO_2 _matrix. Figure 1a shows the plane-view TEM image of the sample annealed at 800°C (sample name: 'RS-1'). Numerous small Ge nanocrystals can be seen to be distributed throughout the film. The average diameter of the nanocrystal is 2.4 nm with the full width at half maxima [FWHM] of the size distribution being 0.8 nm. Figure 1b shows the electron micrograph of the sample annealed at 900°C for 30 min (sample name: 'RS-2'). The Gaussian fitting of the size distribution gives an average nanocrystal size of about 4.3 nm. Figure 1c shows the micrograph of the sample annealed at 1,000°C for 30 min (sample name: 'RS-3'). From TEM micrograph, it is seen that the 'RS-3' sample contains both large- and small-sized nanocrystals, which may occur due to the clustering of nanoparticles during the heat treatment process. A broad distribution in particle size with an average value of 10 nm indicates an increase in size with annealing temperature. The formation of Ge nanocrystals is attributed to the precipitation of Ge within the thermodynamically favorable SiO_2 _layer during post-deposition annealing in N_2_. The crystallization process is a dynamical one with nucleation and growth, in addition to the migration of the Ge nanocrystals. It has been reported [20] that the diffusion of Ge in SiO_2 _and nucleation of Ge depend on the annealing temperature. The size of the nanocrystals increases with increasing annealing temperature due to enhanced nucleation and growth process of Ge nanocrystals at the Si-SiO_2 _interface. Furthermore, a higher annealing temperature leads to an increase in the critical nucleus size, and would also raise the barrier for nucleation.

**Figure 2:**
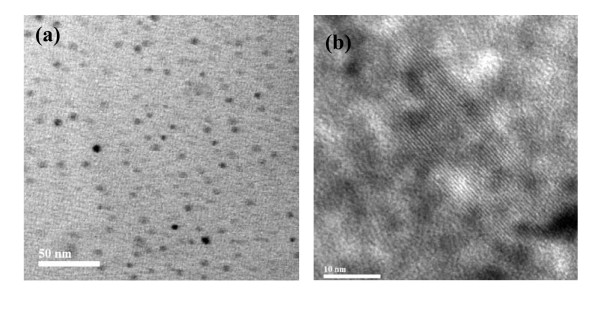
**Plane-view TEM micrographs of Ge-NC**. Embedded in (**a**) Al_2_O_3 _matrix (sample 'RA'), (**b**) HfO_2 _matrix (sample 'RF').

Figure 2a shows the plane-view TEM images of Ge NCs embedded in Al_2_O_3 _matrix annealed at 900°C (sample name: 'RA'). The dark patches are Ge nanocrystals of diameter 5-10 nm in the amorphous Al_2_O_3 _matrix. The nanocrystals are almost spherical and are well dispersed in the Al_2_O_3 _matrix. First, the size distribution of Ge nanocrystals obtained from TEM image analysis has been plotted. Then, the mean nanocrystal size was determined by fitting the distribution curve with the Gaussian distribution function. The estimated size distribution of the nanocrystals for 'RA' sample can be approximated by a Gaussian function with an average diameter of 7.1 nm. The change in Gibbs free energy of formation of GeO (-111.8 kcal/mol) is much smaller than that of high-k Al_2_O_3 _(-378.2 kcal/mol) [21] , which results in the oxidation of Al and agglomeration of Ge atoms into nanocrystals in Al_2_O_3 _matrix during thermal annealing at high temperatures. Figure 2b shows a high-resolution TEM micrograph of Ge nanocrystals embedded in HfO_2 _matrix and annealed at 900°C (sample name: 'RF'), which exhibit clear lattice fringes. The average diameter of the nanocrystal is about 7.8 nm. The change in Gibbs free energy [ΔG] of formation (at 298.15 K) of GeO (-111.8 kcal/mol) [21] is much smaller than that of high-k HfO_2 _(-260.1 kcal/mol). Therefore, the change in Gibbs free energy is negative in the forward direction in the following reaction.

(1) $$\text{Ge}{\text{O}}_{\text{2}}+\text{ Hf }\to \text{ Hf}{\text{O}}_{\text{2}}+\text{ Ge}$$

Hence, the mixture of HfO_2 _and Ge has the lower Gibbs free energy in the co-sputtered film, resulting in the agglomeration of Ge atoms into nanocrystals.

### Photoluminescence characteristics of undoped Ge nanocrystals

**Figure 3:**
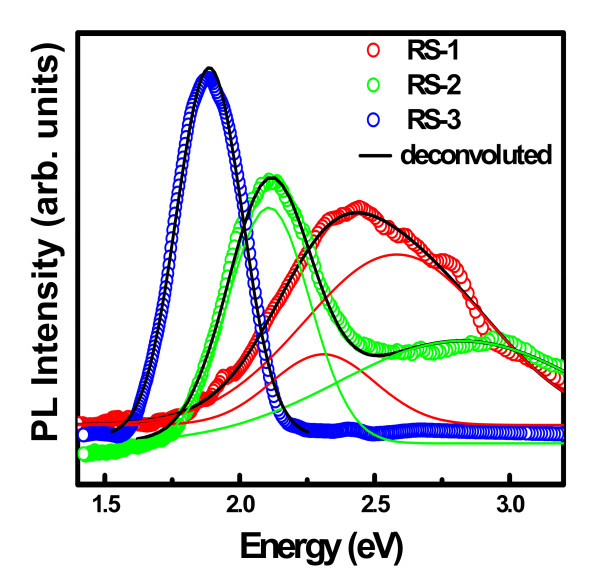
**Room-temperature photoluminescence from Ge nanocrystals embedded in SiO_2 _matrix annealed at different temperatures**.

Figure 3 presents the size-dependent photoluminescence spectra of Ge nanocrystals embedded in SiO_2 _matrix. For a closer insight into the PL results, the spectra have been deconvoluted using Gaussian function as shown in Figure 3, and the results are summarized in Table 2. For 800°C annealed ('RS-1') sample, the PL spectrum can be represented by two peaks centered at 2.31 and 2.58 eV with FWHM of 0.38 and 0.68 eV, respectively. Similarly, for 'RS-2' sample, the PL spectrum consists of the peaks centered at 2.11 and 2.8 eV with FWHM of 0.30 and 0.91 eV, respectively. On the other hand, 'RS-3' sample shows a sharp luminescence at 1.88 eV with FWHM of 0.24 eV. The PL peaks at 2.31, 2.11, and 1.88 eV are observed in accordance to quantum size effect for 'RS-1', 'RS-2', and 'RS-3' samples having average nanocrystal diameters of 2.4, 5.3, and 10 nm, respectively. Hence, the above three PL peaks originate due to radiative recombination of excitons in quantum-confined Ge nanocrystals. Several researchers [8,22,23] have reported size-independent photoluminescence in the energy range of 2.5 to 3.2 eV from Ge nanocrystals, the origin of which is attributed to oxygen vacancies [VO^-^], oxygen-germanium vacancy pairs (VGe, VO)^+^, and related defect centers. Hence, a broad PL from 2.5 to 3.2 eV for as-deposited sample, 2.58 eV PL peak for 'RS-1', and 2.8 eV PL peak for 'RS-2' sample might be related to the defect centers. The PL intensity of defect related peak gets reduced, and that of excitonic recombination is enhanced with the increase of annealing temperature due to improved crystallinity of Ge nanocrystals. However, a red shift of the excitonic peak with increased annealing temperature due to larger-sized nanocrystals manifests the confinement of charge carriers.

**Table 2:** PL peak energy and nanocrystal size for SiO_2 _embedded Ge NCs

Sample name	PL peak details		d_NC_ (TEM) nm
	Peak position (eV)	FWHM (eV)	
'RS-1'	2.31-2.58	0.38-0.68	2.4 ± 0.8
'RS-2'	2.11-2.8	0.30-0.91	5.3 ± 1.3
'RS-3'	1.88	0.24	10 ± 3

d_NC_, nanocrystal diameter; FWHM, full width at half maxima; 'RS-1', 'RS-2', and 'RS-3', sample structures defined in Table 1; PL, photoluminescence; TEM, transmission electron microscopy.

**Figure 4:**
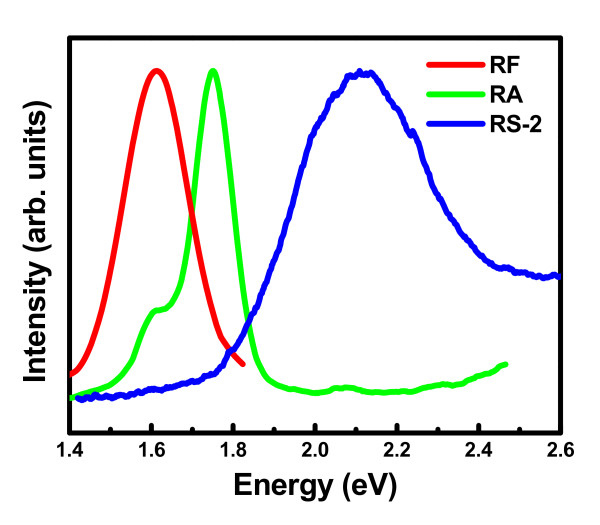
**Room-temperature photoluminescence from Ge nanocrystals embedded in HfO_2_, Al_2_O_3_, and SiO_2 _matrix annealed at 900°C**.

Figure 4 shows the effect of host matrix on room temperature photoluminescence spectra of Ge nanocrystals embedded in SiO_2 _('RS-2'), Al_2_O_3 _('RA'), and HfO_2 _('RF') and annealed at a temperature of 900°C. The PL spectrum for 'RS-2' sample explained in the previous section indicates that the 2.11 eV peak originates due to radiative recombination in quantum-confined Ge nanocrystals. Two intense broad emission peaks are observed around 1.75 eV and 1.67 eV for samples 'RA' and 'RF', respectively. The difference in PL peak energy between the samples may be attributed to the variation in average particle size in combination with the matrix induced effect. In order to interpret the result quantitatively, a simple confinement model [5] has been applied by considering electrons and holes confined independently in quantum dots of radius R

(2) $$E_{nl}=E_{g}+\frac{\hbar^{2}}{2\mu_{e-h}}{\left(\alpha_{nl}/R\right)}^{2}-1.786e^{2}/kR$$

where the second term represents the kinetic energy of electron and holes, and the last term denotes the Coulomb interaction term; *μ*_e-h _is the reduced mass of excitons, *k *is the static dielectric constant (for Ge, *k *= 16.3), *α*_nl _is the eigenvalue of the zeroth-order spherical Bessel function (*α*_10 _= π), and the band gap energy (*E_g_*) of Ge = 0.66 eV. Table 3 presents the calculated Ge nanocrystals size according to quantum confinement model using Equation 2 along with that estimated from TEM micrograph. From the Table 3, it is seen that there is a slight difference in the extracted size from the confinement theory and TEM observations. However, the carriers confined in the quantum dot in this case are under a finite potential, which has not been considered in the present confinement model. Depending upon the host oxide matrix, the conduction and valence band offsets between the germanium nanocrystals and matrix are different, which leads to surrounding matrix-dependent confinement potential [24]. The polarization interface charge-induced nanocrystal band gap modification may also play an important role due to the difference in dielectric constant of the host matrix and the nanocrystals [24]. Therefore, the present study shows an experimental evidence of the role of dielectric constant and band offsets on the optical band gap of Ge nanocrystals bounded in different oxide matrices.

**Table 3:** Size of Ge nanocrystals

Embedded matrix^a^	PL peak energy (eV)	d_NC _[confinement model] (nm)^b^	d_NC _(TEM) (nm)^b^
HfO_2 _('RF')	1.67	7.1	7.8
Al_2_O_3 _('RA')	1.75	6.6	7.1
SiO_2 _('RS-2')	2.12	5.9	5.3

^a^Ge nanocrystals embedded in different oxide matrices and ^b^d_NC_, nanocrystal diameter; PL, photoluminescence; TEM, transmission electron microscopy; 'RF', 'RA', and 'RS-2', sample structures defined in Table 1.

### Emission characteristics of Er-doped Ge nanocrystals

**Figure 5:**
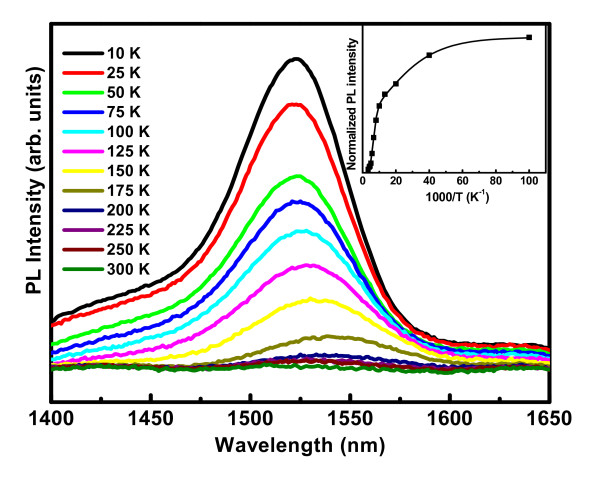
**Temperature-dependent photoluminescence spectra of Er-doped Ge nanocrystals embedded in Al_2_O_3 _matrix**. Inset picture shows the variation of normalized integral intensity of the PL peaks with temperature.

Though visible luminescence is observed from undoped Ge nanocrystals embedded in oxide matrix, rare earth-doped Ge nanocrystals are attractive for emission in fiber optic wavelength (1.54 μm). Er-doped (0.18 wt% Er) Ge nanocrystals (4-7 nm) fabricated by pulsed-laser deposition and annealed at 900°C have been studied for emission in the above wavelength range. Figure 5 shows the temperature-dependent photoluminescence spectra of the Er-doped Ge nanocrystals in Al_2_O_3 _matrix showing the emission peak at 1.54 μm due to the intra-4f ^4^*I*_13/2 _→^4^*I*_15/2 _transition (first excited state to the ground state) of Er atoms. From the figure, it is observed that as the temperature is decreased, the luminescence intensity corresponding to 1.54 μm emission is increased, and the peak is shifted slightly towards the lower wave lengths, indicating the origin of the PL peak to the transitions in Er^3+ ^ions but not in defect states. The inset of the Figure 5 shows the temperature-dependent integrated intensity of the PL peak for 0.18 wt% Er-doped Ge nanocrystals embedded in Al_2_O_3 _matrix annealed at 900°C. The solid line in the inset is fitted using a double exponential function [25],

(3) $$I_{PL}\text{(}T\text{)}=\frac{I_{\text{0}}}{\text{1}+c_{\text{1}}\text{exp}\left(-E_{1}/k_{B}T\right)+c_{\text{2}}\text{exp}\left(-E_{2}/k_{B}T\right)}$$

*I*_0 _being the intensity at absolute zero temperature, *E*_1 _and *E*_2 _are the activation energies, and *c*_1 _and *c*_2 _are the corresponding coupling coefficients. At low temperatures (*T <*75 K), the PL peak intensity is observed to be weakly temperature-dependent, with small thermal activation energy of 5.1 meV. With increase in temperature above 100 K, the PL peak intensity is observed to be quenched with large activation energy of 84.8 meV. It is suggested that the main energy transfer mechanism is the Förster mechanism [26], which is a nonoptical dipole-dipole interaction. Since the Förster mechanism is effective over several nanometres, it is likely that this mechanism is mainly responsible for the energy transfer from Ge nanocrystals to Er^3+ ^ions.

### Electroluminescence characteristics of undoped Ge nanocrystals

**Figure 6:**
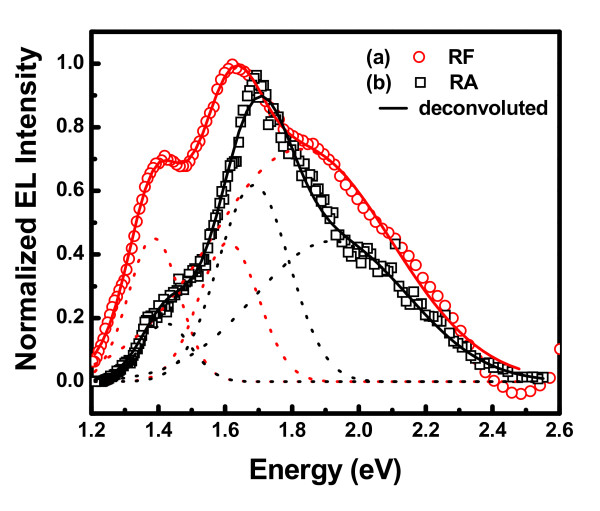
**Room-temperature electroluminescence spectra of MIS structure containing Ge nanocrystals**. Embedded in (**a**) HfO_2 _and (**b**) Al_2_O_3 _matrix.

Electroluminescence characteristics due to the recombination of injected carriers into Ge nanocrystals can be employed to remove any ambiguity about the origin of light emission. MIS structures fabricated on Si with Si/Ge nanocrystals embedded in the dielectric layer [1,27-29] have been widely studied in this regard. A critical challenge for the MIS LED based on nanocrystals embedded in oxide has been the development of a method for efficient carrier injection. Therefore, the electroluminescence characteristics have been studied only for lower band gap high-k oxides. Under the positive gate bias, electron current from the Si conduction band is enhanced using high-k HfO_2_/Al_2_O_3 _as a blocking oxide. For the negative gate bias, the hole injection from the Si valance band can be enhanced, and the electron current from the gate electrode can be suppressed using high-k HfO_2_/Al_2_O_3 _as a blocking oxide. Figure 6 shows the room temperature EL spectra of MIS structure containing Ge nanocrystals embedded in HfO_2 _and Al_2_O_3 _matrices with injected current of 7 mA and 0.11 mA, respectively. The EL spectra are dependent on the polarity of the potential bias, and emission is observed only under a negative gate bias. The spectra show a broad emission in the visible and near infrared region. For HfO_2 _embedded Ge NCs ('RF') device, the EL spectrum can be fitted by three Gaussian peaks (red dotted line) centered at 1.38, 1.61, and 1.82 eV and having FWHM of 0.15, 0.18, and 0.54 eV, respectively. Similarly, the EL spectra of Al_2_O_3 _embedded Ge NCs ('RA') device consist of peaks centered at 1.42, 1.69, and 1.92 eV with FWHM of 0.15, 0.21, and 0.46 eV, respectively. The occurrence of more than one EL peak can be either due to the size distribution of the Ge NCs or to the different recombination mechanisms. From TEM analysis, it is observed that the size distribution is nearly Gaussian shaped. It seems that the second hypothesis is more appropriate in our case. Therefore, the most intense peaks at 1.61 eV for 'RF' sample and 1.69 eV for 'RA' sample are attributed to the electron hole recombination in Ge nanocrystals, in corroboration with photoluminescence results, though a peak shift is observed in EL either due to Stark effect or the sample heating at high injection. There are several reports of luminescence in the blue-green region with the peak energy independent of the size of the nanocrystals [8,29]. Therefore, the observed weaker emission band around 1.82 and 1.92 eV originates due to radiative recombination through defects, which are located at the interface of the nanocrystals [26,29]. The very weak peak at 1.4 eV is attributed to the oxygen related defects in GeO_2_.

### Memory characteristics of Ge nanocrystals in oxide matrices

**Figure 7:**
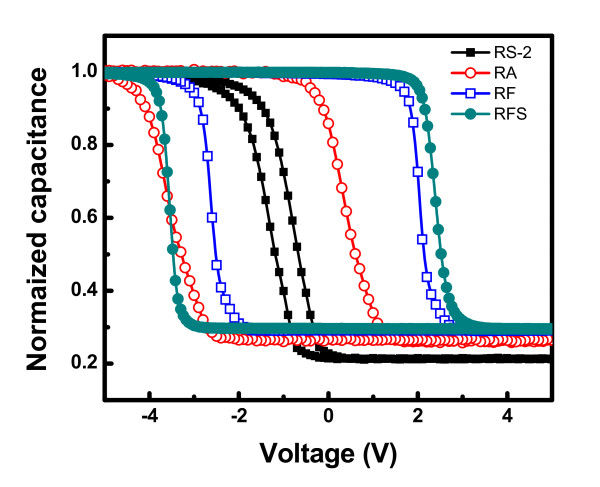
**Capacitance-voltage hysteresis behavior of the MIS structures containing Ge nanocrystals embedded in different dilectric matrices**.

Ge nanocrystals embedded in oxide matrices have potential applications in both flash-memory and light-emitting devices. From the electrical point of view, flash-memory structures with Ge nanocrystals embedded in dielectric layer have been proposed to improve the data retention with faster access speed. For future scaled-down complementary-metal-oxide-semiconductor [CMOS] devices, various high-permittivity (k) dielectric (HfO_2_, Al_2_O_3_) materials have been suggested to replace the SiO_2_. Therefore, the electrical characteristics of new memory structures that are compatible with current CMOS process technology were investigated. Figure 7 presents the high-frequency (1 MHz) capacitance-voltage [C-V] characteristics of the MIS structures fabricated using Ge nanocrystals embedded in different dielectric matrices, for a voltage sweep of ± 7.5 V. The high-frequency C-V characteristics reveal significant hysteresis, indicating the charge storage in Ge nanocrystals. For SiO_2 _embedded ('RS-2') device, a small flat-band voltage shift [ΔV_FB_] of 0.55 V is observed. However, a large Δ*V_FB _*of 3.98, 4.66, and 5.88 V is observed for the Al_2_O_3 _embedded ('RA'), HfO_2 _embedded ('RF') and VARIOT structure (sample name: 'RFS'), respectively. From maximum ΔV_FB_, the stored charge density *N_charge _*has been calculated using [11] the following relation and the results are presented in Table 4.

**Table 4:** Details of memory window and charge retention characteristics of different Ge NCs memory devices

Sample	ΔV_FB _at *t *= 0 (V)	Charge storage (cm^-2^)	ΔV_FB _after 10 years (V)	Charge loss after 10 years (%)
'RS-2'	0.55	3.2 × 10^10^	0.29	47
'RA'	3.98	7.6 × 10^12^	2.67	33
'RF'	4.66	1.6 × 10^13^	2.91	37
'RFS'	5.88	2.1 × 10^13^	4.94	16

**Figure 8:**
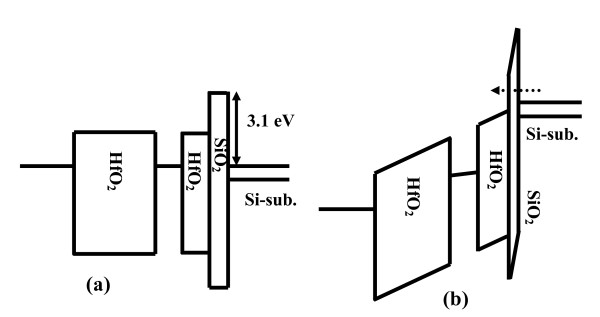
**Schematic energy band diagram of VARIOT memory device**. At (**a**) flat-band condition and (**b**) under program mode.

'RS-2', 'RA', 'RF', and 'RFS', sample structures defined in Table 1; ΔV_FB_, small flat-band voltage shift.

(4) $$N_{charge}=\frac{\Delta V_{FB}}{\frac{q}{\varepsilon_{0}}\left(\frac{t_{CO}}{\varepsilon_{CO}}+\frac{t_{NC}}{\varepsilon_{NC}}\right)}$$

where Δ*V_FB _*is the flat-band voltage shift, *q *is the electronic charge; *t_CO _*and *ε_CO _*are the thickness and relative permittivity of the control oxide; *t_NC _*and *ε_NC _*are the diameter and relative permittivity of the nanocrystal; and *ε_0 _*is the permittivity of the free space. The memory widow is found to be significantly increased for all the high-k oxide (Al_2_O_3 _and HfO_2_) samples as compared to SiO_2 _embedded one ('RS-2') under the same bias sweeping. By using a high-k dielectric in place of SiO_2_, a larger tunneling current is achieved in MIS structure due to the lower electron barrier height of HfO_2 _(1.2 eV) [30] as compared to SiO_2 _(3.1 eV). By using high-k dielectric as a gate oxide, under program mode, the electron current from the Si conduction band is enhanced, and under the erase mode, the hole current from the Si can be increased [15]. Similarly, the electron current from the gate electrode can be suppressed using high-k Al_2_O_3 _or HfO_2 _as a blocking oxide. The VARIOT ('RFS') sample consisting of 2.5-nm thick SiO_2 _followed by 5.0-nm thick HfO_2 _as the tunneling dielectric shows the maximum ΔV_FB_, the reason for which is discussed in the following sentences. Figure 8a, b shows the energy band diagrams including the valence band of the VARIOT structure in flat-band condition and under program mode, respectively. During writing process, when a sufficient voltage occurs across the SiO_2 _film (as shown in Figure 8b), it forces the conduction band edge of the Si substrate to be higher than the conduction band edge of HfO_2_. The tunneling current will then be mainly determined by strong direct tunneling contribution. On the other hand, the tunneling current density of a memory cell with single-layer tunnel HfO_2 _is determined by a much smaller Fowler-Nordheim current. This indicates that the program voltage will be less and the writing speed will be faster for the memory with VARIOT structure than that of a single HfO_2 _tunnel barrier. Due to the asymmetric stack structure of tunneling barrier of VARIOT device, the erasing speed of the VARIOT device may not be as fast as its writing speed.

**Figure 9:**
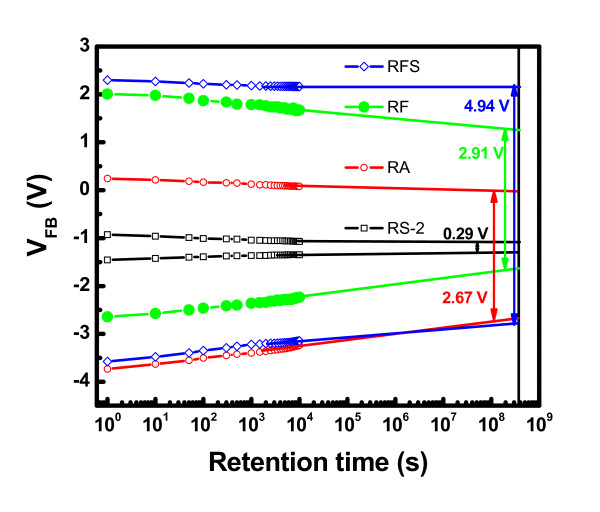
**Retention characteristics of the MIS structures containing Ge nanocrystals embedded in different dielectric matrices**.

Figure 9 shows the retention characteristics of different Ge NCs memory devices at room temperature. At first, the memory capacitor was programmed under a drive gate voltage of +7.5 V for 1 s. Then, the V_FB _was measured with time. Similarly, the memory capacitor was erased under a drive gate voltage of -7.5 V for 1 s, and the V_FB _was measured with time. The initial memory window width (ΔV_FB_) for all the samples is presented in Table 4. Assuming the logarithmic behavior for retention, the extrapolation of V_FB _shift for memory capacitor has been performed up to 10 years. After 10 years of retention, the estimated ΔV_FB _is presented in Table 4. The charge losses of the 'RS-2', 'RA', 'RF', and 'RFS' devices are estimated to be 47%, 33%, 37%, and 16%, respectively after 10 years of retention. The memory device with Al_2_O_3 _matrix shows better charge retention (33% loss) than that of HfO_2 _(37% loss) due to the larger band gap of Al_2_O_3 _(6.8 eV) [31] as a blocking oxide compared to HfO_2 _(5.8 eV) [32]. The VARIOT tunnel barrier memory device shows best retention properties compared to others. This result indicates that the SiO_2 _in the VARIOT structure plays a very important role because of the higher band offset during retention condition.

## Conclusions

In conclusion, we have reported a systematic study on the size- and host matrix-dependent photoluminescence characteristics of Ge nanocrystals showing the origin of visible luminescence due to the quantum confinement of charge carriers. This is corroborated by the broad visible electroluminescence characteristics from devices with Al_2_O_3 _and HfO_2_, attributed to the radiative recombination from Ge nanocrystals and also from the defect states. A two-stage quenching process has been observed from 1.54 μm emission characteristics of Er-doped Ge nanocrystals due to the energy transfer process between Er and Ge following Förster's mechanism. A large memory window of 5.88 V and high retention time of 16% charge loss per 10 years have been obtained in MIS structures using Ge nanocrystals floating gate with VARIOT tunneling barrier.

## Abbreviations

EL: electroluminescence; FWHM: full width at half maxima; HRTEM: high-resolution transmission electron microscopy; NCs: nanocrystals; PL: photoluminescence; QDs: quantum dots; SEM: scanning electron microscopy; VARIOT: variable oxide thickness; V_FB_: flat-band voltage.

## Competing interests

The authors declare that they have no competing interests.

## Authors' contributions

SD prepared the Ge nanocrystals embedded in different dielectric matrix. RA prepared the Er-doped Ge nanocrystals. SD, RA, and SM performed the treatment of experimental data and calculations. SD, RA, and SKR prepared the manuscript initially. SKR, LP, RKS, and AD conceived of the study and participated in its design and coordination. All the authors read and approved the final manuscript.
